# Cytokine-driven PANoptosis of alveolar macrophages mediated by STAT1 underlies acute lung injury in hypervirulent *Klebsiella pneumoniae* infection

**DOI:** 10.1128/mbio.03958-25

**Published:** 2026-03-24

**Authors:** Qi Xu, Xiaoxuan Liu, Heng Heng, Han Wang, Wenxing Zhao, Mei Luo, Guan Yang, Mingxiu Peng, Edward Wai-Chi Chan, Sheng Chen

**Affiliations:** 1 Department of Biology and Genetics, The College of Life Sciences and Health, Wuhan University of Science and Technology 47900, Wuhan, China; 2 Department of Food Science and Nutrition, Faculty of Science, The Hong Kong Polytechnic University 26680, Hong Kong, China; 3 Department of Infectious Diseases and Public Health, Jockey Club College of Veterinary Medicine and Life Sciences, City University of Hong Kong 53025, Hong Kong, China; 4 State Key Lab of Chemical Biology and Drug Discovery, Department of Applied Biology and Chemical Technology, The Hong Kong Polytechnic University, Hong Kong, China; Universiteit Gent, Gent, Belgium

**Keywords:** *Klebsiella pneumoniae*, alveolar macrophage, PANoptosis, cytokine, STAT1

## Abstract

Understanding the fundamental mechanisms that govern innate immune and inflammatory responses during *Klebsiella pneumoniae* (*Kp*) infection is critical for developing effective therapeutic strategies against this public health threat. We found that hypervirulent *Kp* (hv*Kp*) infection led to a significant depletion of alveolar macrophages (AMs) in mice. Single-cell RNA sequencing (scRNA-Seq) analyses suggested that this loss was associated with the activation of cell death signaling pathways. Further investigation confirmed that hv*Kp* infection simultaneously activated key executioners of pyroptosis, apoptosis, and necroptosis, indicating the induction of PANoptosis, a synergistic cell death process. Cell-cell communication analysis revealed significant enhancement of pro-inflammatory signaling pathways, including TNF and IFN-II, in infected lungs. Mechanistically, cytokines produced during infection, particularly IFN-γ in combination with others, synergistically induced PANoptosis in macrophages. The key transcription factor STAT1 was significantly activated upon infection. STAT1-deficient mice exhibited markedly reduced AM loss, bacterial burdens, tissue damage, and mortality following hv*Kp* challenge, alongside impaired activation of key PANoptotic effectors. Furthermore, STAT1 deficiency indirectly regulated inflammatory death in AMs by impairing IFN-γ production in immune cells like NK cells. In summary, this study reveals that hv*Kp* infection triggers STAT1-dependent PANoptosis in AMs, leading to immune cell death, organ failure, and high mortality, providing new insights into the pathogenesis of severe *Kp* infections.

**IMPORTANCE:**

*Klebsiella pneumoniae*, particularly hypervirulent strains (hv*Kp*), poses a critical public health threat due to its capacity to cause severe, rapidly progressing infections such as pneumonia and sepsis, often leading to acute lung injury (ALI) and high mortality. Despite the recognized role of excessive inflammation and cytokine storms in hv*Kp* pathogenesis, the precise mechanisms linking immune hyperactivation to fatal tissue damage remain poorly defined. This study reveals that hv*Kp* infection triggers a coordinated form of inflammatory cell death, PANoptosis, in AMs, the frontline immune defenders in the lungs. We identify the transcription factor STAT1 as a central regulator of this process, driven by a synergistic cytokine milieu, especially involving IFN-γ. Our findings establish a direct mechanistic pathway from hv*Kp*-induced cytokine release to STAT1-mediated PANoptosis, macrophage depletion, and subsequent lung failure. This work not only advances the understanding of hv*Kp* virulence but also highlights host signaling pathways and specific cytokines as potential therapeutic targets to modulate immunopathology and improve outcomes in severe *Klebsiella* infections.

## INTRODUCTION

*Klebsiella pneumoniae* (*Kp*) is a Gram-negative, capsulated bacterium and an opportunistic pathogen. It is responsible for causing a spectrum of diseases in the host organism and is considered a major pathogen for nosocomial pneumonia and sepsis (1). Notably, *Kp* accounts for about one-third of all Gram-negative infections (2) and 5–20% of sepsis cases induced by Gram-negative bacteria (3). However, therapeutic options for *Kp* infections are limited with the emergence of carbapenem-resistant *Kp* (CR-*Kp*) by acquiring exogenous antibiotic resistance genes like *bla*_KPC-2_, *bla*_VIM_, or *bla*_NDM-1_, rendering them resistant to carbapenems and various other antibiotics, which raises a global public health threat. Beyond its resistance to multiple antimicrobials, *Kp* possesses sophisticated strategies for defending the immune responses of the host, including molecular mimicry, immune modulation, and biofilm formation (4). These mechanisms enable *Kp* to avoid detection and elimination by the host, often resulting in excessive activation of the immune system (5, 6). However, this hyperactivation can lead to the overproduction of pro-inflammatory cytokines, such as interleukin (IL)−1β and tumor necrosis factor (TNF)-α, both of which are directly associated with lung injury and multiple organ failure (7).

Acute lung injury (ALI) is a clinical syndrome caused by various pathogenic factors, including acute pneumonia, sepsis, severe trauma, and acute pancreatitis. It is initially characterized by pulmonary inflammation and increased microvascular permeability, which can progress to acute respiratory distress syndrome (ARDS) (8). The lungs are particularly susceptible to injury during sepsis, and the primary risk factors of ALI in >50% patients were attributed to sepsis (9). However, the pathophysiology and pathogenesis of *Kp*-induced ALI remain incompletely understood. As the most abundant immune cells in the lung under homeostatic conditions (10), macrophages play a crucial role in the development of sepsis-induced ALI. Alveolar macrophages (AMs) account for nearly 95% of leukocytes in the airways and are the first immune cells to encounter pulmonary pathogens (11, 12). For this reason, their response to infection can critically influence the outcomes of infectious diseases (11, 13). In this scenario, emerging evidence has revealed that AM death plays an important role in determining the pathogenesis of pulmonary infections.

Previous studies have demonstrated a close association between PANoptosis and lung injury (14, 15). PANoptosis has been elucidated in a variety of conditions ranging from normal development to microbial infectious, autoimmune diseases, cytokine storm, and cancer (16). For example, during SRAS-CoV-2 infection, patients often develop systemic symptoms of varying severity, with PANoptosis contributing to the exacerbation of pulmonary cytokine storms and the progression of COVID-19 associated pneumonia to ARDS (17–19). *Kp* infection has been reported to activate multiple programmed cell death pathways, including pyroptosis (20), apoptosis (21), and necroptosis (22), which collectively contribute to disease severity and immune response regulation. Therefore, understanding the interplay between cell death mechanisms and ALI is essential for developing effective therapies against *Kp* infections. Recently, we demonstrated that the high mortality associated with hv*Kp* is largely due to the cytokine storm induced by this pathogen (6). Given the link between severe inflammation, cell death processes, and tissue damage, as well as the lack of detailed understanding of this connection in hv*Kp* infection, it is essential to identify the mechanism underlying tissue damage pathways involved in the host defense against *Kp* and to elucidate their roles in *Kp* pathogenesis.

## RESULTS

### Lung tissue damage is associated with AMs PANoptosis in hv*Kp* infection

**Fig 1:**
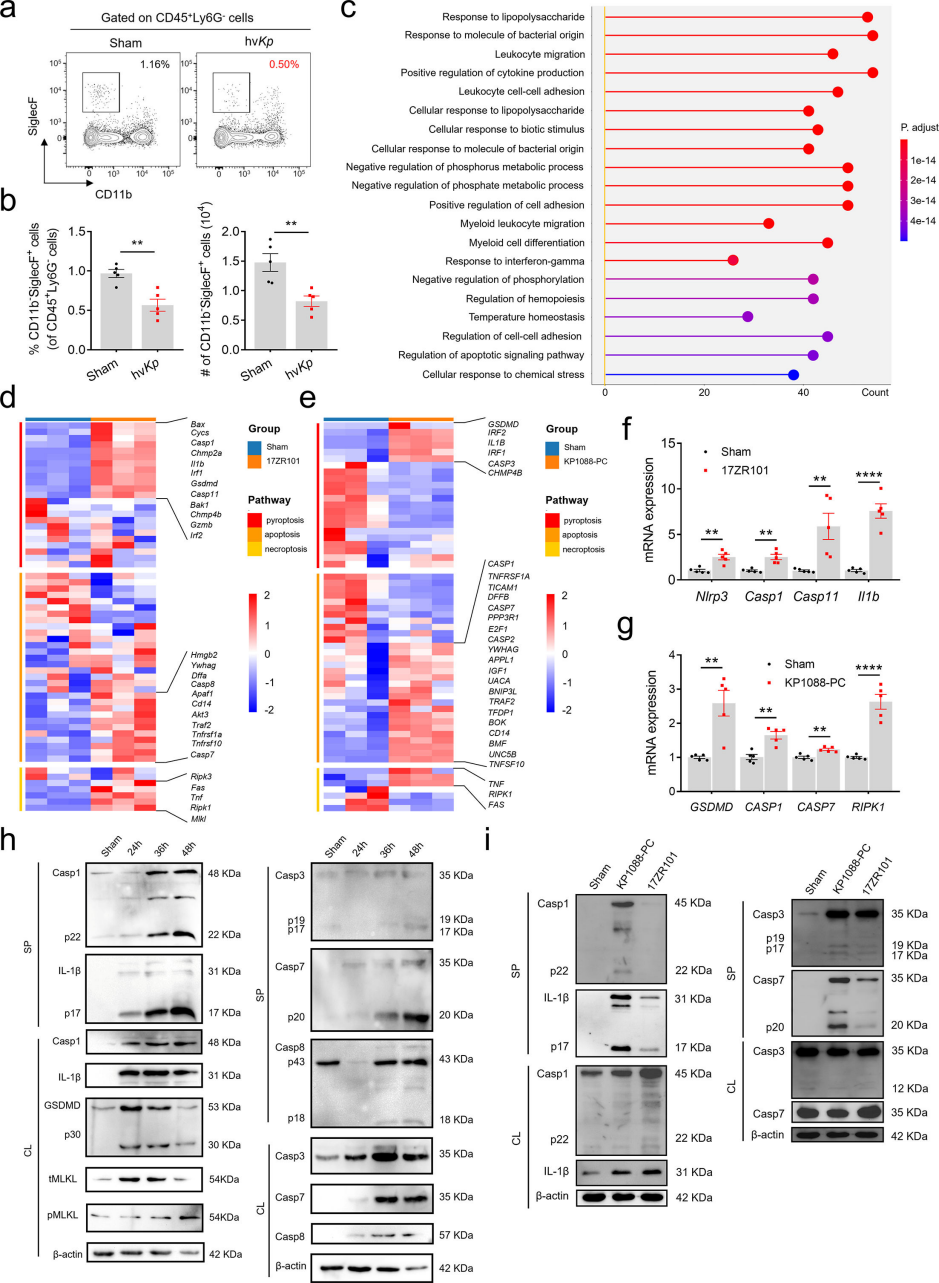
AMs encountered PANoptosis in *Kp* infection. (**a**) Representative bivariate flow cytometry plots showing CD11b and SiglecF expression on live CD45^+^Ly6G^−^ cells obtained from matched lung samples. (**b**) Quantification of SiglecF^+^CD11b^−^ cells in lungs of sham- and hv*Kp*-treated mice. *n* = 5/group. (**c**) Go enrichment analysis of changing pathways in AMs. (**d and e**) Heatmap deciphering the expression level of marker genes of pyroptosis, apoptosis, and necroptosis in *Kp*-infected or sham-treated lungs (**d**), sham- or *Kp*-treated THP-1 cell (**e**). (**f and g**) Fold change in expression level of genes related to PANoptosis markers in lung cells (**f**) and THP-1 cells (**g**). *n* = 5/group. (**h and i**) Immunoblot analysis of pro- and Casp1, pro- and IL-1β, GSDMD, total MLKL and phosphorylated MLKL, pro- and Casp3, pro- and Casp7, pro- and Casp8, and β-actin in BMDMs cells at 24, 36, and 48 hpi and THP-1 (**i**). SP, supernatant. CL, cell lysis. Data are represented as mean ± SEM. **P < 0.01, ****P < 0.0001.

Understanding the host immune response to hv*Kp* infection is critical for elucidating the molecular mechanisms underlying severe *Kp* pathogenesis. We previously demonstrated that the tissue damage and high mortality associated with hv*Kp* infection is largely due to a cytokine storm induced by the pathogen. Single-cell RNA sequencing (scRNA-seq) revealed a significant reduction in AMs in hv*Kp*-infected mice (6). As lung-resident macrophages, AMs possess high phagocytic activity and express pattern recognition receptors (PRRs), enabling them to respond effectively to bacterial and fungal pathogens (6). Then, we proposed that the lung tissue damage in hv*Kp* infection was correlated with AMs deduction. To validate this hypothesis, we performed flow cytometry analysis to assess AM populations in sham- and hv*Kp*-infected mice. Consistent with the scRNA-seq data, hv*Kp* infection led to a marked depletion of AMs (SiglecF^+^CD11b^−^) in the lungs (Fig. 1a and b).

Gene ontology (GO) enrichment analysis revealed that AMs from *Kp*-infected lungs exhibited significant changes in pathways related to responses to lipopolysaccharide and bacterial components, regulation of cytokine production, myeloid cell differentiation, response to interferon (IFN)-γ, and apoptotic signaling (Fig. 1c). Reclustering analysis identified 12 AM subclusters (Fig. S1a), all of which were reduced in hv*Kp*-infected lungs (Fig. S1b). Notably, the regulation of apoptotic signaling pathways was enriched in 8 out of 12 subclusters, suggesting that most AMs underwent apoptosis during hv*Kp* infection (Fig. S1c). Collectively, these findings suggest that AM deficiency may result from inflammatory cell death during *Kp* infection.

Transcriptomic analysis of lung tissues from sham- and hv*Kp*-infected mice (Bioproject accession no.: PRJNA851242) showed that hv*Kp* infection upregulated genes associated with pyroptosis (*Casp1*, *Casp11*, *Gsdmd*), apoptosis (*Casp7*, *Casp8*), and necroptosis (*Ripk1*, *Mlkl*), indicating activation of all three cell death pathways (Fig. 1d). Similarly, RNA-seq analysis of *Kp*-infected THP-1 cells (PRJNA1195673) also indicated inflammatory cell death (Fig. 1e), which was further validated by RT-qPCR (Fig. 1f and g). Given the crosstalk among these pathways, we propose that hv*Kp* triggers PANoptosis—a synergistic cell death process regulated by molecules such as caspase-3, caspase-8, and RIPK.

Upon infection, the cleaved form of GSDMD, a key executor of pyroptosis, was detected (Fig. 1h). Cleavage by caspase-1 enables GSDMD to form membrane pores, resulting in cell swelling and pyroptosis (23). Consistent with canonical inflammasome activation, we observed caspase-1 activation and IL-1β release from 24 h post-infection (hpi) (24). Additionally, apoptosis was evidenced by cleavage of caspase-3, -7, and -8, while phosphorylated MLKL indicated necroptosis. The activation of these pathways increased in a time-dependent manner during infection (Fig. 1h) and was corroborated in human THP-1 cells infected with hv*Kp* strain 17ZR101 (Fig. 1i). Together, these results indicate that *Kp* infection induces AM deficiency via PANoptosis.

### *Kp* infection alters cell-cell communications in AMs

**Fig 2:**
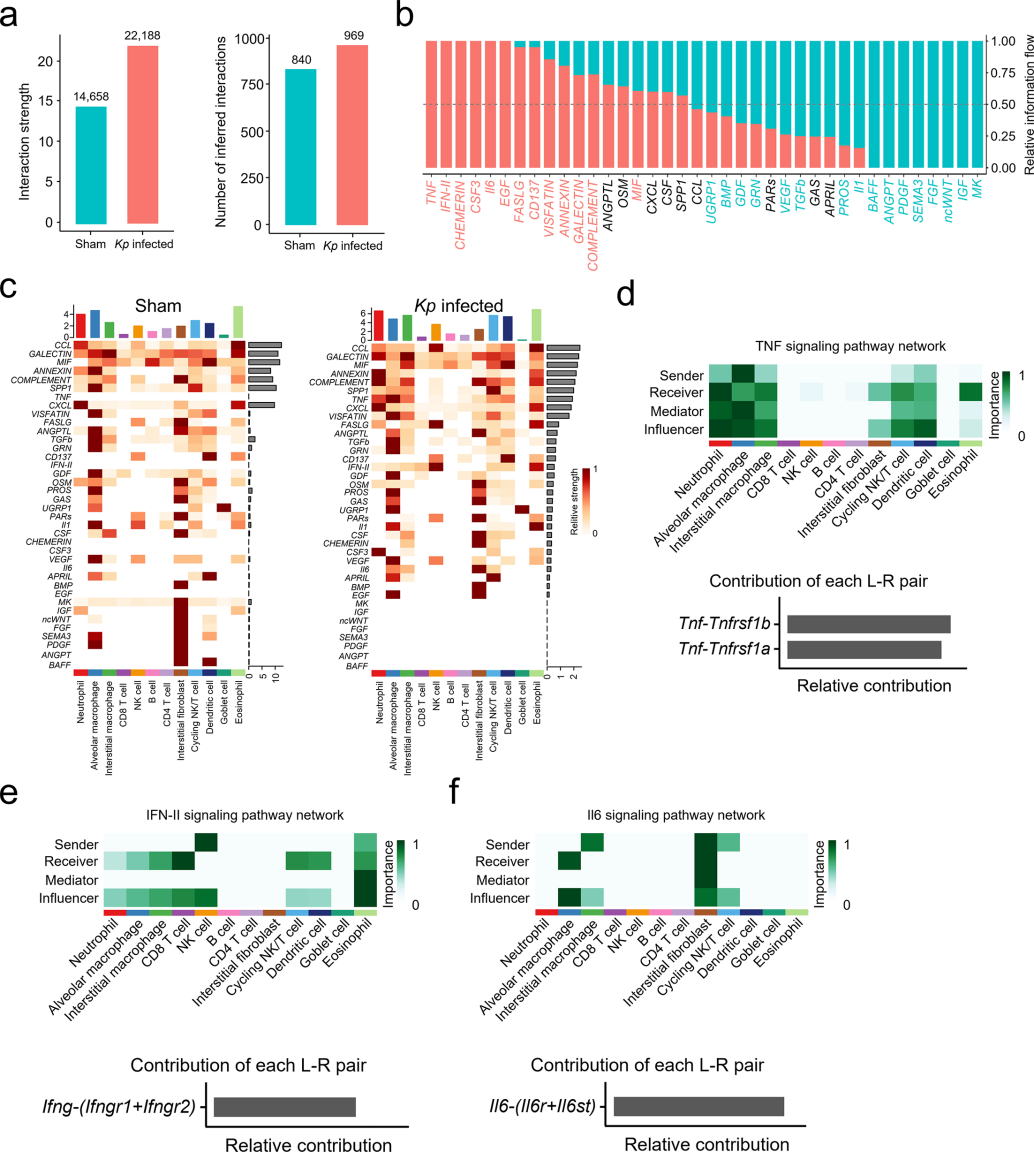
Cellular communication in *Kp*-infected lungs. (**a**) The interaction weights/strength and the number of interactions in sham and *Kp*-infected samples. (**b**) Comparison of the overall information flow of each signaling pathway or ligand-receptor pair in sham- and *Kp*-treated lungs. (**c**) Identification of signaling changes across distinct conditions using quantitative contrasts. The right row bar indicates the sum of outgoing signaling strength across all cell groups for one pathway. The top row bar height is the sum of outgoing signaling strength across all pathways for one cell group. (**d**) The heatmap shows the relative importance of each cell group based on the computed four network centrality measures of the TNF signaling network and relative contribution of each ligand-receptor pair to the overall communication network of the TNF signaling pathway, which is the ratio of the total communication probability of the inferred network of each ligand-receptor pair to that of the TNF signaling pathway. (**e**) The inferred IFN-II signaling network and relative contribution of each IFN-II ligand-receptor pair. (**f**) The inferred Il6 signaling network and relative contribution of each Il6 ligand-receptor pair.

To investigate potential interactions between AMs and other immune cells during hvKp infection, we performed CellChat analysis (25) on our previously published GEO data set (GSE220594) (6). This unbiased ligand-receptor–based approach revealed significant alterations in both the strength and the number of cell-cell interactions in hv*Kp*-infected lungs compared to sham controls (Fig. 2a). The identified ligand-receptor pairs among 12 cell types were categorized into 37 signaling pathways, including CCL, GALECTIN, MIF, ANNEXIN, COMPLEMENT, SPP1, TNF, and CXCL pathways. Of these, 18 pathways showed notable changes in the hv*Kp*-infected group compared to sham (Fig. 2b). Notably, pro-inflammatory pathways, such as TNF, IFN-γ, and IL-6, were enriched in hv*Kp*-infected lungs (Fig. 2c).

CellChat further predicted that AMs, along with neutrophils and interstitial macrophages (IMs)—both known sources of TNF ligands—significantly contribute to TNF signaling during hv*Kp* infection. The TNF signaling network was found to be complex and highly redundant, with multiple ligand sources targeting a large proportion of myeloid cells. Among all ligand-receptor pairs, TNF signaling was dominated by the *Tnf* ligand and its multimeric *Tnfrsf1a*/*Tnfrsf1b* receptors (Fig. 2d). Additionally, most IFN-II interactions among lung cells were paracrine, with NK cells and eosinophils as major senders, and neutrophils, AMs, IMs, CD8 T cells, and eosinophils as major recipients. IFN-II signaling was dominated by the *Ifng* ligand and its *Ifngr1*/*Ifngr2* receptors (Fig. 2e). For IL-6 signaling, IMs, interstitial fibroblasts, and cycling NK/T cells were the main sources, while AMs and interstitial fibroblasts were the primary recipients. Activation of CHEMERIN/CSF3/EGF signaling in *Kp*-infected lungs and MK/IGF/PDGF signaling in sham-infected lungs also exhibited cell type-specific features (Fig. S2). Collectively, these findings suggest that *Kp* infection induces distinct signaling pathway activation, with robust pro-inflammatory cytokine signaling occurring in AMs.

### Inflammatory cytokines elevated in *Kp*-infected mice are positively correlated with cell death

**Fig 3:**
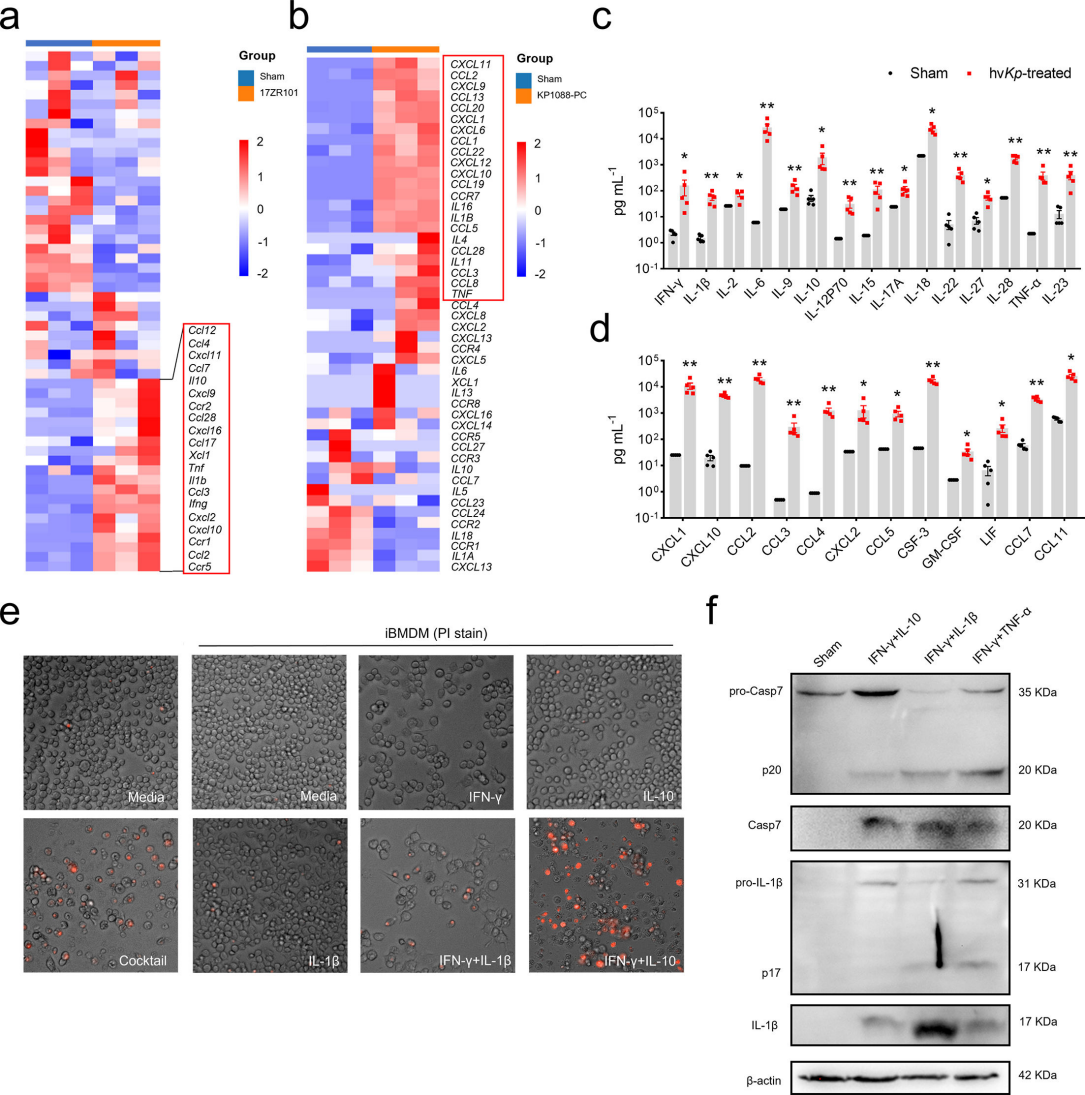
IFN-γ is a dominant cytokine in induction of inflammatory cell death. (**a and b**) Heatmap showed the differentially expressed genes in lung samples of mice and THP-1 cells with *Kp* infection or sham control, highlighting the expression levels of cytokines, chemokines, and their receptor genes. (**c and d**) Profiling of serum cytokine and chemokine levels of *Kp*-infected mice or sham control. (**e**) Representative images of iBMDMs stimulated with cytokine cocktail, mock control, and IFN-γ, TNF-α, and IL-10 individually or by combination at 36 h. (**f**) Immunoblot analysis of pro-(P35) and cleaved caspase-7, pro- (P31) and cleaved IL-1β in mock or iBMDM treated with IFN-γ and IL-10, IFN-γ and IL-1β, or IFN-γ and TNF-α. Actin was used as the internal control. Data are represented as mean ± SEM. *P < 0.05, **P < 0.01.

Previous findings showed that TNF-α and IFN-γ played prominent roles in organ damage by inducing inflammatory cell death (15), and that TNF-α and IFN-β secreted by COVID-19–infected cells could trigger PANoptosis in bystander cells (26), providing evidence of the multiple roles of these cytokines in PANoptosis. We have also reported that excessive inflammatory cytokines are associated with tissue damage and high mortality of hv*Kp* infection (6). We next questioned whether the cytokines induced by hv*Kp* infection are uniquely involved in programmed cell death in AMs. Analysis of published transcriptomic data from hv*Kp*-infected lungs revealed that genes encoding cytokines, chemokines, and their receptors—such as *Cxcl11*, *Il10*, *Il1b*, and *Ccr2*—were highly upregulated (Fig. 3a), a finding also observed in *in vitro* infection models (Fig. 3b). To further characterize the dominant cytokines and chemokines during *Kp* infection, we profiled serum from sham- and hv*Kp*-infected mice. Of 36 cytokines and chemokines measured, 27 were significantly upregulated in hv*Kp*-infected mice compared to controls (Fig. 3c and d); eight were undetected; and only CXCL5 showed no significant difference (data not shown). Circulating levels of IFN-γ, IL-1β, IL-6, IL-15, IL-18, TNF-α, and chemokines, such as MCP-1, CXCL10, CCL3, and CCL4, were most elevated in hv*Kp*-infected mice.

To assess the ability of these cytokines to induce cell death, we treated immortalized BMDMs (iBMDMs) with cytokines highly upregulated during *Kp* infection. Treatment with a cocktail of IFN-γ, TNF-α, IL-10, IL-6, and IL-1β induced robust cell death within 36 h (Fig. 3e), whereas individual cytokines did not induce significant cell death. Testing various combinations, we found that co-stimulation with IFN-γ and IL-10 or IFN-γ and IL-1β triggered cell death at levels comparable to the full cytokine cocktail, suggesting a synergistic effect (Fig. 3e). After 36 h, high levels of cell death were observed, accompanied by morphological changes, such as cell rounding, swelling, and nuclear condensation. To test whether the increased lethality and organ failure observed during *Kp* infection could be attributed to robust induction of inflammatory cell death, we characterized the activation of effector proteins in cytokine-treated iBMDMs. Treatment with combinations of IFN-γ and IL-10, IL-1β, or TNF-α led to increased activation of PANoptotic molecules, such as caspase-7 and IL-1β (Fig. 3f). These findings indicate that cytokine combinations induce inflammatory cell death, mirroring the symptoms of *Kp* infection. Collectively, our results demonstrate that cytokines produced during *Kp* infection, particularly IFN-γ, drive cell death, tissue damage, and poor prognosis.

### Hv*Kp* infection-driven cell death and lethality are dependent on STAT1-mediated PANoptosis

**Fig 4:**
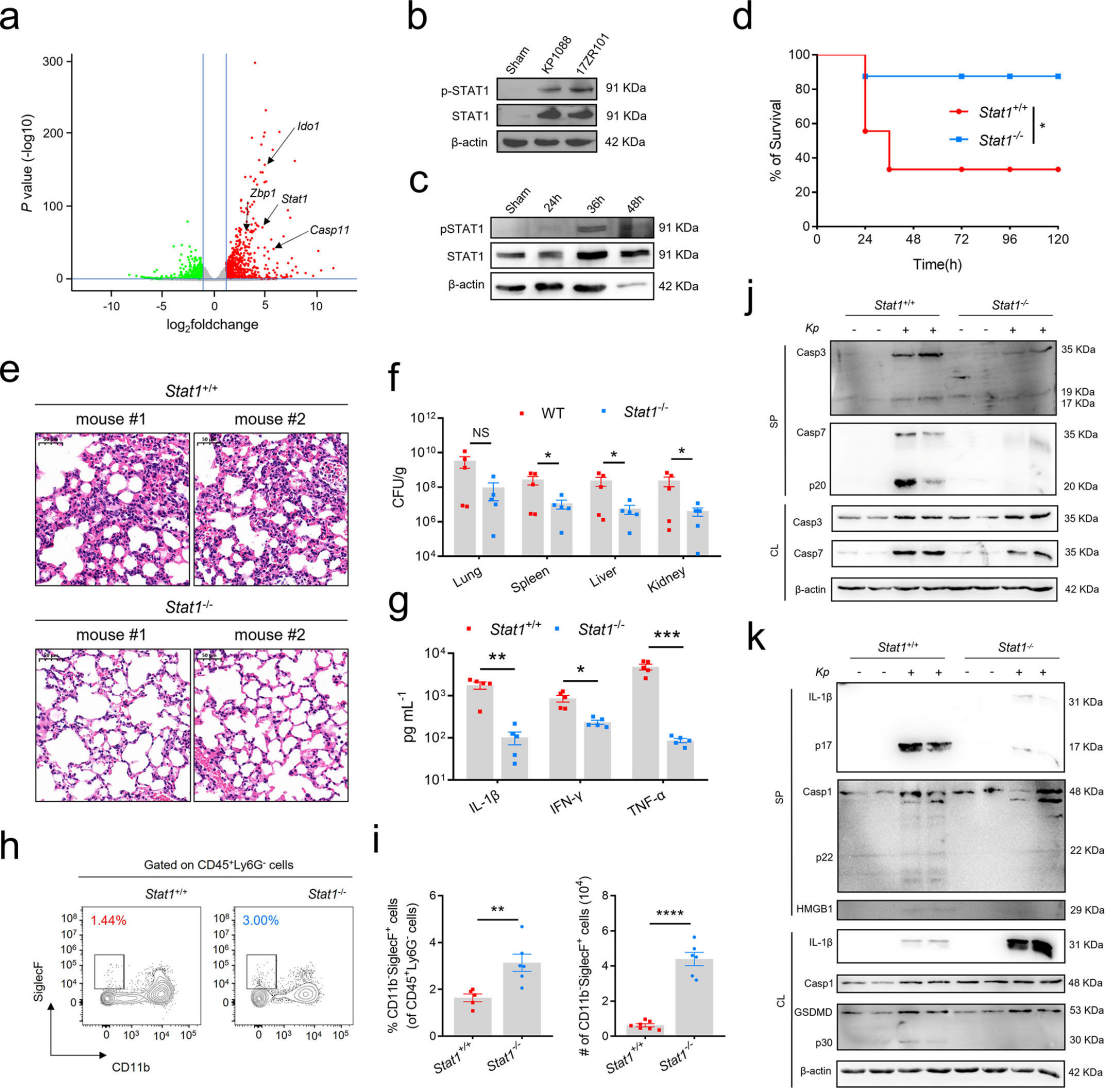
STAT1 deficiency improves survival and prevents PANoptosis induced by *Kp* infection (**a**) Volcano plot of RNA-Seq gene counts in total lung cells obtained from Sham- and *Kp*-infected mice. (**b**) THP-1 cells were infected with *Kp,* and the cell lysate was collected at 12 hpi. The expression level of phosphorylated STAT1 was detected by immunoblotting with β-actin serving as loading control. (**c**) BMDMs were infected with *Kp,* and the cell lysate was collected at the indicated time point. The expression level of phosphorylated STAT1 was detected by immunoblotting with β-actin serving as loading control. (**d**) Survival rate of *Stat1*^+/+^ and *Stat1*^−/−^ mice with *Kp* infection. *n* = 7. (**e**) Representative Hematoxylin and eosin (H&E) staining of lung tissues from *Kp*-infected *Stat1*^+/+^ and *Stat1*^−/−^ mice (scale bar: 50 μm). (**f**) Organ bacteria burden in *Kp*-infected *Stat1*^+/+^ and *Stat1*^−/−^ mice at 12 hpi. (**g**) Quantification of serum IL-1β, IFN-γ, and TNF-α levels from *Kp*-infected *Stat1*^+/+^ and *Stat1*^−/−^ mice by ELISA. (**h and i**) Representative flow cytometric plots and quantification of SiglecF^+^CD11b^-^ cells collected from the lungs of *Kp*-infected *Stat1*^+/+^ and *Stat1*^−/−^ mice. (**j and k**) Immunoblot analysis of pro- and caspase-3, pro- and caspase-7, pro- and IL-1β, pro- and caspase-1, HMGB1, and GSDMD in the BMDMs isolated from *Stat1*^+/+^ and *Stat1*^−/−^ mice that were Sham-treated or *Kp*-infected. β-Actin was used as loading control. SP, cell supernatant. CL, cell lysate. Data is representative of at least three independent experiments. SP, supernatant. CL, cell lysis. Data are represented as mean ± SEM. NS, not significant, *P < 0.05, **P < 0.01, ***P < 0.001, ****P < 0.0001.

STAT1 is essential for IFN signaling and plays a critical role in defense against both viral and bacterial infections (27). Transcriptomic analysis revealed that *Stat1*, along with *Jak1* and *Jak2*, was highly upregulated in *Kp*-infected mice compared to controls (Fig. 4a; Fig. S3). As a master transcription factor, STAT1 orchestrates multiple transcriptional programs and facilitates crosstalk between distinct signaling cascades (28). Consistent with RNA-seq data, we observed significant upregulation of STAT1 and its phosphorylated form (p-STAT1) in *Kp*-infected THP-1 cells (Fig. 4b) and BMDMs (Fig. 4c), with phosphorylation detectable only in infected cells (6).

To determine whether STAT1 drives AM PANoptosis and organ failure during *Kp* infection, we infected wild-type (WT) and *Stat1*^−/−^ mice with the K1 hv*Kp* strain KP1088-PC intraperitoneally. This route of infection was used for survival studies to model the systemic dissemination and sepsis commonly associated with severe hv*Kp* infections, which reliably lead to acute lung injury and high mortality, allowing us to investigate the underlying mechanisms of organ failure. Approximately 70% of WT mice succumbed within 36 h, whereas lethality decreased to 10% in *Stat1*^−/−^ mice (Fig. 4d). At 12 hpi, STAT1-deficient mice exhibited significantly reduced proinflammatory cell infiltration in lungs (Fig. 4e) and bacterial burdens in the lung, liver, spleen, and kidney compared to WT mice (Fig. 4f). Importantly, STAT1-deficient mice showed decreased serum IFN-γ levels (Fig. 4g) and increased AM numbers in the lungs (Fig. 4h and i) following *Kp* challenge. These results suggest that inflammatory cell death of AMs and subsequent organ failure during *Kp* infection are, at least in part, regulated by STAT1.

Our findings indicate that PANoptosis occurs in response to *Kp* infection and may contribute to organ failure and lethality. Then, we investigated whether STAT1 is required for PANoptosis activation during *Kp* infection. *Stat1*^−/−^ BMDMs exhibited impaired activation of PANoptotic effectors in response to *Kp* infection. Immunoblot analysis revealed that activation of caspase-7 and caspase-1—key mediators of apoptosis and pyroptosis, respectively—was almost abolished in STAT1-deficient BMDMs (Fig. 4j and k). Extracellular HMGB1, a DAMP released during various forms of cell death (29), was detected in the supernatant of *Kp*-infected WT BMDMs but not in STAT1-deficient cells (Fig. 4k).

### STAT1 deficiency reverses IFN-γ production in NK cells

**Fig 5:**
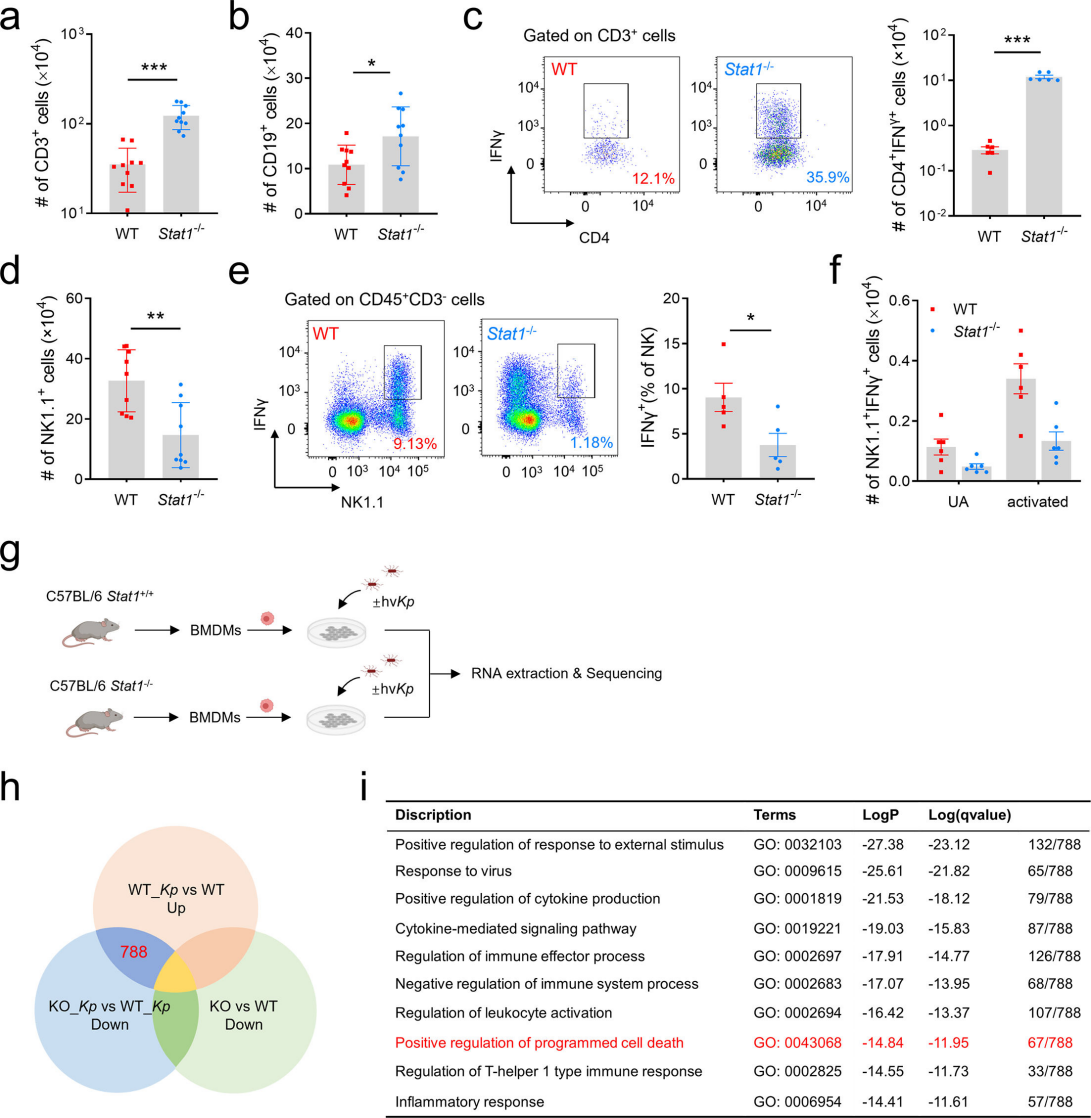
Absence of STAT1 alleviated *Kp*-induced lymphopenia while leading to NK cell dysfunction. (**a and b**) Total lung cells isolated from *Kp*-infected WT and *Stat1*^−/−^ mice were cultured with the activation cocktail and subjected to IFN-γ intracellular staining. The number of T cells (**a**) and B cells (**b**) recovered from the lungs of *Stat1*^+/+^ or *Stat1*^−/−^ mice with *Kp* infection. *n* = 10. (**c**) Representative dot plots showing percentages of CD4^+^IFN-γ^+^ within CD3^+^ cells and representative bar diagrams showing the number of CD4^+^IFN-γ^+^ T cells in *Stat1*^+/+^ and *Stat1*^−/−^ mice with *Kp* infection. (**d**) The number of NK cells recovered from the lungs of *Stat1*^+/+^ or *Stat1*^−/−^ mice with *Kp* infection. (**e and f**) Representative dot plots showing percentages of NK1.1^+^IFN-γ^+^ within CD45^+^CD3^-^ cells and representative bar diagrams showing the number of IFN-γ^+^ NK cells in *Stat1*^+/+^ and *Stat1*^−/−^ mice with *Kp* infection. (**g**) Experimental design for RNA-Seq of BMDMs with or without *Kp* infection. (**h**) Analysis of the overlap in genes identified by RNA-seq data as being differentially expressed in *Stat1*^+/+^ and *Stat1*^−/−^ BMDMs in response to *Kp* infection. (**i**) Gene ontology analyzes 788 genes from (**h**). The 67 genes enriched in positive regulation of programmed cell death as being differentially expressed between these four groups are listed in Table S1. Data are represented as mean ± SEM. *P < 0.05, **P < 0.01, ***P < 0.001.

T cells lacking STAT1 are resistant to activation-induced cell death (30). Post-mortem analyses of spleens and lymph nodes from COVID-19 patients have revealed a lack of germinal centers, which are essential for generating high-quality, long-term antibody responses (31). It is possible that cytokine signaling exacerbates lymphopenia through direct lymphocyte killing. We previously reported a significant decrease in T cells (CD3^+^) and B cells (CD19^+^) in *Kp*-infected lungs, with T cell reduction attributed to inhibited proliferation and increased apoptosis (32), a hallmark of severe infectious disease (15). Here, we found that STAT1 deletion restored immune cell compartments in *Kp*-infected mice. Recovery of T cells (Fig. 5a) and B cells (Fig. 5b) in infected lungs was observed in *Stat1*^−/−^ mice. Since impaired cytokine production is characteristic of lymphopenia, we examined T cell responses in infected WT and *Stat1*^−/−^ mice. While only 12.1% of CD3^+^ T cells from WT *Kp*-infected lungs were CD4^+^IFN-γ^+^, *Stat1*^−/−^ mice exhibited a significant increase in both frequency and number of CD4^+^IFN-γ^+^ T cells (Fig. 5c). Interestingly, flow cytometry also revealed a drastic reduction in NK cell numbers in STAT1-deficient mice compared to WT upon *Kp* infection (Fig. 5d). As NK cells are potent producers of chemokines and cytokines, particularly IFN-γ (33), we observed impaired IFN-γ production in NK cells from *Stat1*^−/−^ mice (Fig. 5e and f), consistent with reduced serum IFN-γ levels (Fig. 4h). Thus, STAT1 deficiency impairs IFN-γ production in NK cells, further alleviating inflammatory PANoptosis in AMs (Fig. 4f and g).

Although we identified a critical role for STAT1 in promoting inflammatory cell death during *Kp* infection, we speculated that STAT1 is not the direct regulator of PANoptosis. To better understand the regulatory network, we analyzed differentially expressed genes in *Stat1*^−/−^ BMDMs with or without *Kp* infection (Fig. 5g). Integrative RNA-seq analysis identified 788 genes upregulated in *Kp*-infected *Stat1*^+/+^ BMDMs and downregulated in *Stat1*^−/−^ BMDMs (Fig. 5h). GO analysis revealed enrichment in cytokine production, cytokine-mediated signaling, and positive regulation of programmed cell death. Of these, 67 genes were associated with positive regulation of programmed cell death (Fig. 5i), including direct executors of PANoptosis, such as *Casp1*, *Casp7*, *Casp8*, *Casp11*, and *Ripk3*, further confirming the crucial role of STAT1 in *Kp*-induced PANoptosis.

Interestingly, we also identified six transcriptional regulators—NR3C1, IRF1, SP110, DDIT3, EGR1, and PLAGL2—that may act downstream of STAT1 in inflammatory cell death (Table S1). Notably, IRF1 has been shown to regulate cell death, including PANoptosis (15), in other contexts. SP100 has been implicated in genotoxicity and cell death via caspase activation (34), while DDIT3 promotes RIPK1 activation and necroptosis (35), as well as osteoblast pyroptosis via NLRP3/caspase-1/GSDMD (36). Together, these results confirm that STAT1 is a key upstream regulator of *Kp*-induced PANoptosis, contributing to *Kp*-mediated pathology and lethality.

## DISCUSSION

Although we have demonstrated that the cytokine storm induced multiple organ failure is the main reason of host death during hv*Kp* infection (6), the link between excessive cytokine production and organ failure remains unclear. Emerging evidence has revealed that AM cell death plays important roles in influencing the progression of lung inflammation (37, 38). There is also increasing recognition that inflammation and cell death reciprocally affect each other and form an auto-amplification loop of these two factors, which in turn exaggerates inflammation (39). In this study, we established the critical role of PANoptosis of AMs in *Kp*-induced lung inflammation and high mortality (Fig. 1). Recent studies in other severe infections suggest that PANoptosis can promote coagulopathy, such as disseminated intravascular coagulation (DIC) (40, 41), which contributes to multi-organ dysfunction (15, 26). Therefore, pharmacological manipulation of AMs' death may represent a potential therapeutic avenue for ALI secondary to *Kp* infection. While our study demonstrates the concurrent activation of pyroptosis, apoptosis, and necroptosis effectors, defining the precise quantitative contribution of each death subroutine to overall AM loss and lung injury requires further investigation. Future experiments utilizing specific pharmacological inhibitors (e.g., caspase-1 for pyroptosis, Z-VAD for apoptosis) in combination with our *in vitro* and *ex vivo* models could help dissect their individual roles.

IFN-γ is primarily produced by T lymphocytes, particularly CD4^+^ and CD8^+^ T cells, as well as NK cells upon viral infections. In this study, we demonstrated that the combination of IFN-γ with TNF-α/IL-10/IL-1β induced upregulation and activation of key molecules in PANoptosis. Although studies have reported that PANoptosis could not be induced by IFN-γ alone, it can only occur with the addition of TNF-α to IFN-γ (15, 42). We and other groups have observed minimal cell death with IFN-γ treatment alone (43) (Fig. 3e). We found that, apart from IFN-γ and TNF-α, the combination of IFN-γ and IL-10/IL-1β synergistically induced cleavage of pro-IL-1β and caspase-7 (Fig. 3f), suggesting that IFN-γ and IL-10/IL-1β together sensitize the cells to undergo PANoptosis. While NF-κB activation generally drives pro-survival signaling (44), the combination of IL-1β/TNF-α with IFN-γ is cytotoxic. Although the different cytokine combinations have been reported to trigger cells to undergo PANoptosis, all of them suggest that cytokine blockade using inhibitors could be effective in anti-inflammatory therapy. Treatment with the combination of TNF-α- and IFN-γ-neutralizing antibodies provides protection against SARS-CoV-2 infection lethality in the mouse model (15). Our study suggested that novel therapy targeting specific cytokines, e.g., through inhibition of IFN-γ and IL-10 signaling, might be beneficial for patients suffering from *Kp* infection, and that so-called “cytokine therapy” could be applicable in the scenario of *Kp* infection. Future studies employing pharmacological inhibitors of the JAK/STAT pathway or neutralizing antibodies against cytokines such as IFN-γ and IL-10 in hv*Kp*-infected mice will be crucial for validating these targets and assessing their therapeutic potential.

IFN-γ signals mainly through the JAK/STAT pathway to achieve transcriptional activation of IFN-γ-inducible genes (45). Dysregulation of JAK/STAT signaling is recognized as a major contributor to various diseases, including IBD. We identified a critical role for STAT1 in promoting inflammatory cell death. Several inhibitors of STAT1 and its upstream regulator JAK are in clinical trials for various diseases, and some anti-JAK therapeutics are already clinically available (46, 47). It is important to note that the protective effects observed in whole-body Stat1-deficient mice likely result from a combination of cell-intrinsic loss of STAT1 signaling within AMs and broader systemic effects on cytokine production and immune cell activation. Future studies employing conditional knockout models targeting STAT1 specifically in macrophages or other myeloid cells would be valuable to precisely dissect the cell-intrinsic contributions of STAT1 to PANoptosis initiation versus its role in regulating the cytokine milieu. Our data indicate that STAT1 deficiency impacts the pathogenic cascade at multiple nodes. It impairs the production of key cytokines like IFN-γ (particularly from NK cells), and it directly hampers the activation of PANoptotic executioners within AMs. The present study cannot fully delineate the relative contribution of each mechanism to the overall protective phenotype. It is plausible that the reduced cytokine milieu and the intrinsic resistance to cell death act synergistically to confer survival advantage.

Downstream of STAT1, IRF1 has been identified as critical for inflammatory cell death in response to TNF-α and IFN-γ. IRF1 has previously been shown to regulate cell death in other studies, including the induction of PANoptosis to suppress colorectal tumorigenesis (48, 49). Apart from IRF1, we also identified another five transcriptional regulators that might function in inflammatory cell death. Our integrative transcriptomic analysis identified several transcriptional regulators (e.g., IRF1, SP110, DDIT3) as potential downstream effectors of STAT1 in the PANoptosis pathway. Although these factors have been implicated in cell death regulation in other contexts, their specific roles in hv*Kp*-induced AMs PANoptosis remain to be functionally validated. Future studies involving siRNA or CRISPR-mediated knockout of these candidates in macrophages will be essential to map the precise STAT1-dependent transcriptional network governing inflammatory cell death during *Kp* infection. Although SP110 and DDIT3 have been reported to regulate cell death, how these regulators function in PCD still remains for further investigation.

**Fig 6:**
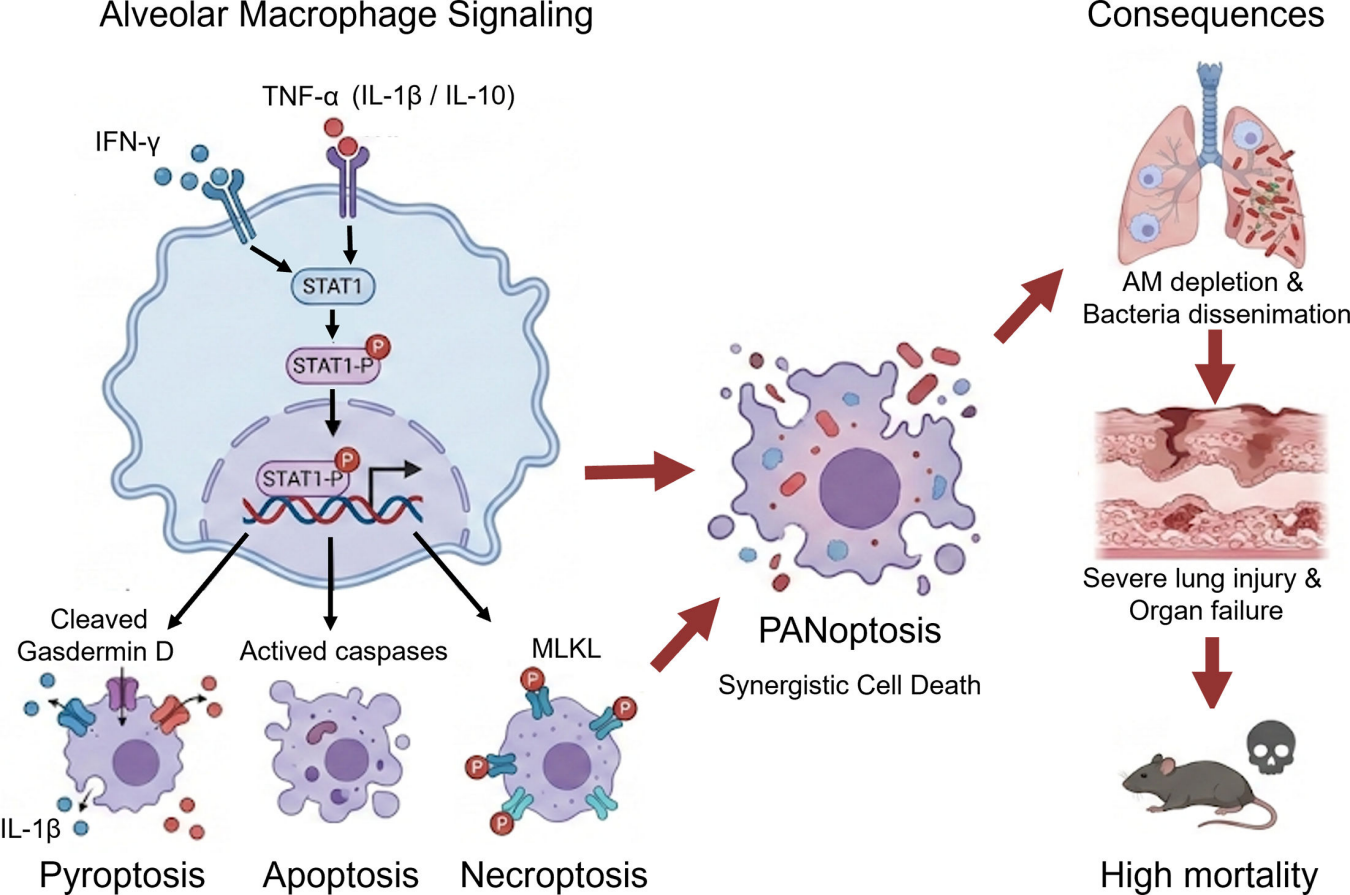
Mechanism of hv*Kp*-Induced PANoptosis in AMs. Hypervirulent Klebsiella pneumoniae (hv*Kp*) infection in the lungs triggers a robust immune response. This leads to the release of pro-inflammatory cytokines, specifically IFN-γ and TNF-α, into the alveolar microenvironment. These cytokines synergistically activate the JAK-STAT1 signaling pathway within AMs. Upon activation, phosphorylated STAT1 translocates to the nucleus, driving the transcriptional upregulation of key cell death regulators. This results in the simultaneous activation of pyroptosis (via GSDMD), apoptosis (via Caspases), and necroptosis (via MLKL), a convergent cell death process known as PANoptosis. The induction of PANoptosis causes massive depletion of alveolar macrophages, impairing bacterial clearance and leading to severe lung injury, organ failure, and high mortality.

Overall, our findings suggested that deletion of STAT1 could reduce the high mortality rate and pathogenic disorders caused by *Kp* infection by obstructing the process of cell death. These findings highlight the importance of maintaining a well-balanced immune response. It may seem contradictory in the normal sense, but weakening the response of the immune system can be a better strategy to fight against *Kp* infections. How to manipulate the host response using immune therapies to achieve a better outcome in confronting *Kp* infections remains a novel treatment strategy. In addition, we established the critical role of STAT1 in *Kp*-induced PANoptosis, filling the unmet understanding of the underlying mechanism of *Kp* pathogenesis (Fig. 6). Our research establishes a critical role for STAT1 and provides a mechanistic foundation for developing host-directed therapeutic strategies. The potential efficacy of JAK/STAT inhibitors or specific cytokine blockade in mitigating hv*Kp*-induced immunopathology warrants further preclinical investigation.

## MATERIALS AND METHODS

### Animal experiments

Six- to eight-week-old animals of both sexes were used in this study. WT C57BL/6 and *Stat1*^−/−^ (C57BL/6Smoc-*Stat1^em2Smoc^*, NM-KO-200611) mice were originally obtained from Shanghai Model Organisms Center, Inc., and housed and bred under specific pathogen-free conditions. WT littermate mice, referred to as WT mice, were used as controls for *Stat1*^−/−^ mice.

### Generation of primary bone marrow-derived macrophages

Bone marrow-derived macrophages (BMDMs) were generated by flushing one femur with 3 mL of PBS. BM cells were then placed into culture in petri dish with BMDM media (DMEM base, 10% FBS, 1× Anti-Anti [Gibco, #15240-062], 1× sodium pyruvate [Gibco, #11360-070], and 1× L-glutamine [Gibco, #25030-081]) containing 50 ng/mL of M-CSF (Peprotech, #315-02-10UG) at 37°C with 5% CO_2_. For all BMDM studies, BMDMs were generated from sex-matched mice as mentioned above. On day 7 of culture, adherent cells were washed three times with sterile 1× DPBS (Gibco, #14190-136) and isolated via treatment with 0.25% trypsin (Gibco, #25200-056) for 3 min at 37°C before mechanical scraping. Cells were counted via hemocytometer before being seeded overnight for functional assessment.

### Cell culture

Primary bone marrow-derived macrophages (BMDMs) from wild-type or *Stat1*^−/−^ mice were isolated following the procedure described by Toda et al. (50). BMDMs were cultured in Dulbecco’s modified Eagle medium (DMEM) (Gibco, #11965092) supplemented with 10% fetal bovine serum (FBS) (Gibco, #A5670201) and 1% penicillin-streptomycin (Gibco, #15140122). BMDMs were seeded into 6-well plates or 24-well plates after 6 days of cultivation and incubated overnight before use. Human monocytes THP-1 (ATCC, #TIB-202) grew in RPMI1640 tissue culture medium (Gibco, # 31800022) supplemented with 10% FBS, 1% nonessential amino acid (Gibco, #11140035), and 1% penicillin-streptomycin at 37°C in a humidified 5% CO_2_ atmosphere. THP-1 cells were differentiated into macrophages by being treated with 150 ng/mL phorbol 12-myristate 13-acetate (PMA, Sigma-Aldrich, #P1585) for 48 h.

### Cell stimulation

BMDMs were stimulated with the following concentrations of cytokines where indicated, unless otherwise noted: 20 ng/mL of IL-6 (Peprotech, #212-16), 20 ng/mL of 20 ng/mL of IL-1β (Peprotech, #211-11B), 25 ng/mL of TNF-α (Peprotech, #315-01A), 50 ng/mL of IFN-γ (Peprotech, #315-05), or 50 ng/mL of IL-10 (Peprotech, #210-10).

### Real-time imaging for cell death

iBMDMs (5 × 10^5^ cells/well) were seeded in 24-well tissue culture plates. Cells were treated with the indicated cytokines and stained with propidium iodide (PI; Sigma-Aldrich, #P4170-1G) at 36-hour post-treatment following the manufacturer’s protocol. PI-positive dead cells are marked with a red mask for visualization.

### *In vivo* infection

Six- to eight-week-old age- and sex-matched wild-type or *Stat1*^−/−^ mice were used for infections. Log-phased K1-*Kp* strain KP1088-PC was adjusted to OD = 0.3 in PBS and diluted to achieve 2.5 × 10^6^ CFU per mL. Mice were inoculated with 5 × 10^5^ *Kp* in 200 µL PBS via the intraperitoneal route. The survival and body weight of mice were monitored for 120 h after infection.

### Cell-cell communication analysis

To explore the cell-cell communications across the 12 cell types, the CellChat (v1.0.0) R package was used for systematic analysis of cell-cell communication (25). We manually updated CellChatDB and performed analysis with standard workflow to predict major incoming and outgoing signals across the signaling pathways by using compute-CommunProbPathway function. Then, we employed the netAnalysis_signaling Role_heatmap function to visualize the signaling strength in each cell type. Specifically, only cell types with more than 10 cells were used to infer cell-cell communication.

### Immunoblot analysis

BMDMs or THP-1 cells were seeded in 6-well plates for immunoblot analysis. Cells were washed twice with PBS and then infected with the *Kp* strain KP1088-PC or 17ZR101 in DMEM or RPMI media without FBS at MOI = 20 for 2 h at 37°C. After that, cells were washed once with PBS and replenished with media containing 300 μg/mL amikacin to remove extracellular bacteria. The infected cells were then incubated at 37°C, and the protein samples were collected at the indicated time points (24, 36, and 48 hpi). The protein was resolved by standard 12% polyacrylamide gels and transferred to polyvinylidene difluoride membranes (Millipore) using the Trans-Blot Turbo Transfer System (Bio-Rad). Membranes were blocked with 5% skim milk in Tris-buffered saline with 0.1% Tween 20 (TBST) at room temperature with shaking and then incubated overnight at 4°C with primary antibody (1:2,000) diluted in 5% skim milk. Primary antibodies used in this study are anti-GSDMD (Abcam, #ab209845), anti-IL-1β (cell signaling technology, CST, #12507), anti-cleaved-IL-1β (CST, #63124), anti-caspase-1 (CST, #24232), anti-cleaved-caspase-1 (CST, #89332), anti-HMGB1 (CST, #6893), anti-caspase-3 (CST, #14220), anti-cleaved-caspase-3 (CST, #9664), anti-caspase-8 (CST, #4927), anti-cleaved-caspase-8 (CST, #8592), anti-caspase-7 (CST, #9492T), anti-cleaved-caspase-7 (CST, #9661T), anti-RIP3 (CST, #3493), anti-phospho-RIP3 (CST, #91702), anti-MLKL (CST, #37705), anti-phospho-MLKL (CST, #37333), anti-STAT1 (CST, #9172T), anti-phospho-STAT1 (CST, #7649T), and anti-β-actin (Abcam, #ab119716). On the second day, the membranes were washed four times with TBST and incubated for 1 h at room temperature with horseradish peroxidase (HRP)–conjugated secondary antibodies (1:5,000 diluted in 5% skim milk; CST anti-rabbit IgG, HRP-linked antibody, #7074; anti-mouse IgG, HRP-linked antibody #7076). After four washes, the protein was developed with UltraSens Pico ECL Kit (MACROLL, 723J111) and imaged with Invitrogen iBright 1500 Imaging System.

### Serum collection and cytokine/chemokine analysis

Blood was collected from the test mice by orbital bleeding and subjected to centrifugation at 10,000 × *g* for 25 min at 4°C to obtain the serum. The serum was stored immediately at −80°C for further analysis. The serum collected from *Kp*-infected mice was diluted 1:10 or 1:100 for cytokine analysis. The level of cytokines and chemokines was determined using ProcartaPlex Mouse Cytokine & Chemokine Panel 1A, 36-plex (Invitrogen, #EPX360-26092-901), following the manufacturer’s instructions.

### Preparation of single-cell suspension from the lung and spleen

Cells collected from different tissues of the test animals were subjected to flow cytometry analysis. Cells from the spleens were obtained by mashing the organ through a 70 µm cell strainer and resuspended in a tube containing RPMI 1640 medium supplemented with 5% fetal bovine serum. To prepare lung cells, lung tissues were excised and incubated in HBSS containing 1× HEPES and collagenase type I at 37°C for 45 min with shaking. The tissue fragments were forced through a 70 µm strainer as described above. Red blood cells were lysed with ACK lysing buffer (Gibco, #A10492-10). Cells were collected by centrifugation at 400 × *g* for 5 min at 4°C and resuspended in FACS buffer for further analysis. The number of cells recovered from organs was counted using Invitrogen Countess 3.

### Flow cytometry analysis

Dead cells were excluded from the analysis by PI or Live/Dead Ghost Dye Violet 510 (Tonbo Biosciences, #13-0870-T100) in flow cytometry experiments. Cell suspensions were washed with FACS staining buffer and incubated at 4°C for 30 min with the following antibodies: PE anti-CD45 antibody (#103106), BV421 anti-CD11b antibody (#101235), APC-Cy7 anti-CD11b antibody (#101225), PerCP/cyanine5.5 anti-Ly6G antibody (#127615), APC anti-CD11c antibody (#117310), BV510 anti-Ly6C antibody (#128033), APC anti-CD3e antibody (#100235), APC/cyanine7 anti-CD19 antibody (#115529), APC/cyanine7 anti-F4/80 antibody (#157315), BV421 anti-CD4 antibody (#100437), PE/cyanine7 anti-NK1.1 antibody (#156513), and PE/Cyanine7 anti-CD170 (Siglec-F) antibody (#155527), and appropriate isotype controls were obtained from Biolegend. Cells were incubated with antibodies for 30 min at 4°C protected from light, washed with FACS buffer, and subjected to analysis using a BD FACSSymphony A1 flow cytometer (BD Bioscience). The acquired data were analyzed by the FlowJo software (Version 10.0.7, Treestar, Palo Alto, CA). For intracellular antibody measurement, cells were incubated with Cell Activation Cocktail (BioLegend, #423303) at 37°C in the dark for 4 h. Anti-IFN-γ-FITC antibody (eBioscience, #12-7177-81) was used to determine the intracellular expression of IFN-γ.

### Measurement of bacterial burden in various organs of the test animals

Tenfold dilutions of homogenized tissue were prepared and spread onto LB agar plates to determine the bacterial load in different organs of the infected animals. The number of bacteria recovered was counted and presented as the number of CFU g^−1^ tissue.

### Real-time quantitative PCR analysis

Real-time quantitative PCR was performed by using a QuantStudio 7 Pro Real-Time PCR System, following the manufacturer’s instructions. Primers used in qPCR are listed in Table S2. cDNA samples were tested in duplicates, and the relative amount of mRNA in different samples was determined by the comparative threshold cycle (ΔΔCT) method, using the glyceraldehyde-3-phosphate dehydrogenase gene (*Gapdh*) for normalization.

### RNA-Seq analysis

Total RNA was extracted from mock- or *Kp*-infected BMDMs collected at 12 hpi by homogenization in TRIzol reagent (Invitrogen, #15596026), followed by chloroform extraction and isopropanol precipitation. DNA was removed by TURBO DNA-free Kit (Invitrogen, #AM1907). RNA samples were sent to Novogene (Hong Kong) Company Limited for sequencing. Routine procedures of mRNA purification and library generation were performed by Novogene. Sequencing reads alignment was performed by using HISAT2. Count-aligned reads and quantification were calculated based on exon regions using FeatureCounts. Significantly changed genes (FPKM ≥ 1 in either WT-, KO-, WT_*Kp*-, or KO_*Kp*-group, |log2fold change| > 1, padj < 0.01) were identified by DESeq2 analysis. Gene set enrichment analysis was performed for gene ontology enrichment analysis.

### Histopathological analysis

The fixed tissues were embedded in paraffin and cut into serial sections (5 mm thick). After dewaxing with xylene and dehydration with ethanol, the samples were stained with hematoxylin and eosin (H&E) and examined microscopically.

### Statistical analysis

Statistical analysis of data obtained in this work was performed using Graphpad Prism 6.0 (GraphPad Software, www.graphpad.com). Statistical analyses on normally distributed data sets were performed using one-way ANOVA with Tukey’s correction for multiple comparisons. The log-rank test was used for comparing survival rates in animal experiments. *P* values < 0.05 were considered significant. Unless otherwise indicated, the survival curve of mice in animal experiments and the results of flow cytometry analysis were representative of at least two independent experiments.

## Data Availability

The raw RNA-seq data has been deposited in the NCBI database under the accession number PRJNA1330535.

## SUPPLEMENTAL MATERIAL

Supplemental Figures mbio.03958-25-s0001.docx Figures S1 to S3.

Table S1 mbio.03958-25-s0002.xlsx Genes associated with positive regulation of programmed cell death revealed by RNA-Seq.

Table S2 mbio.03958-25-s0003.docx Primers for qPCR in this study.

ASM does not own the copyrights to Supplemental Material that may be linked to, or accessed through, an article. The authors have granted ASM a non-exclusive, world-wide license to publish the Supplemental Material files. Please contact the corresponding author directly for reuse.
