# Supplementary material for: Cytokine-driven PANoptosis of alveolar macrophages mediated by STAT1 underlies acute lung injury in hypervirulent Klebsiella pneumoniae infection
Source: mBio. 2026 Mar 24;17(5):e03958-25. doi: 10.1128/mbio.03958-25 (PMC13170300; doi:10.1128/mbio.03958-25)
Supplement: Supplemental Figures — Figures S1 to S3. [file mbio.03958-25-s0001.docx]

***Supplementary Figures for***

**Cytokine-driven PANoptosis of alveolar macrophages mediated by STAT1 underlies acute lung injury in hypervirulent *Klebsiella pneumoniae* infection**

Qi Xu^1, 2#^, Xiaoxuan Liu^2, 3#^, Heng Heng^2^, Han Wang^2, 3^, Wenxing Zhao^2^, Mei Luo^2^, Guan Yang^3^, Mingxiu Peng^4^, Edward Wai-Chi Chan^3, 4^, Sheng Chen^3, 4*^

^1^Department of Biology and Genetics, The College of Life Sciences and Health, Wuhan University of Science and Technology, Wuhan, China

^2^Department of Food Science and Nutrition, Faculty of Science, The Hong Kong Polytechnic University, Hung Hom, Hong Kong

^3^Department of Infectious Diseases and Public Health, Jockey Club College of Veterinary Medicine and Life Sciences, City University of Hong Kong, Kowloon, Hong Kong

^4^State Key Lab of Chemical Biology and Drug Discovery, Department of Applied Biology and Chemical Technology, The Hong Kong Polytechnic University, Hung Hom, China

^#^ These two authors contributed equally to this work.

^*^ Corresponding author: Sheng Chen, The Hong Kong Polytechnic University, Hung Hom, Hong Kong; Email: [sheng.chen@polyu.edu.hk](mailto:sheng.chen@polyu.edu.hk).

**
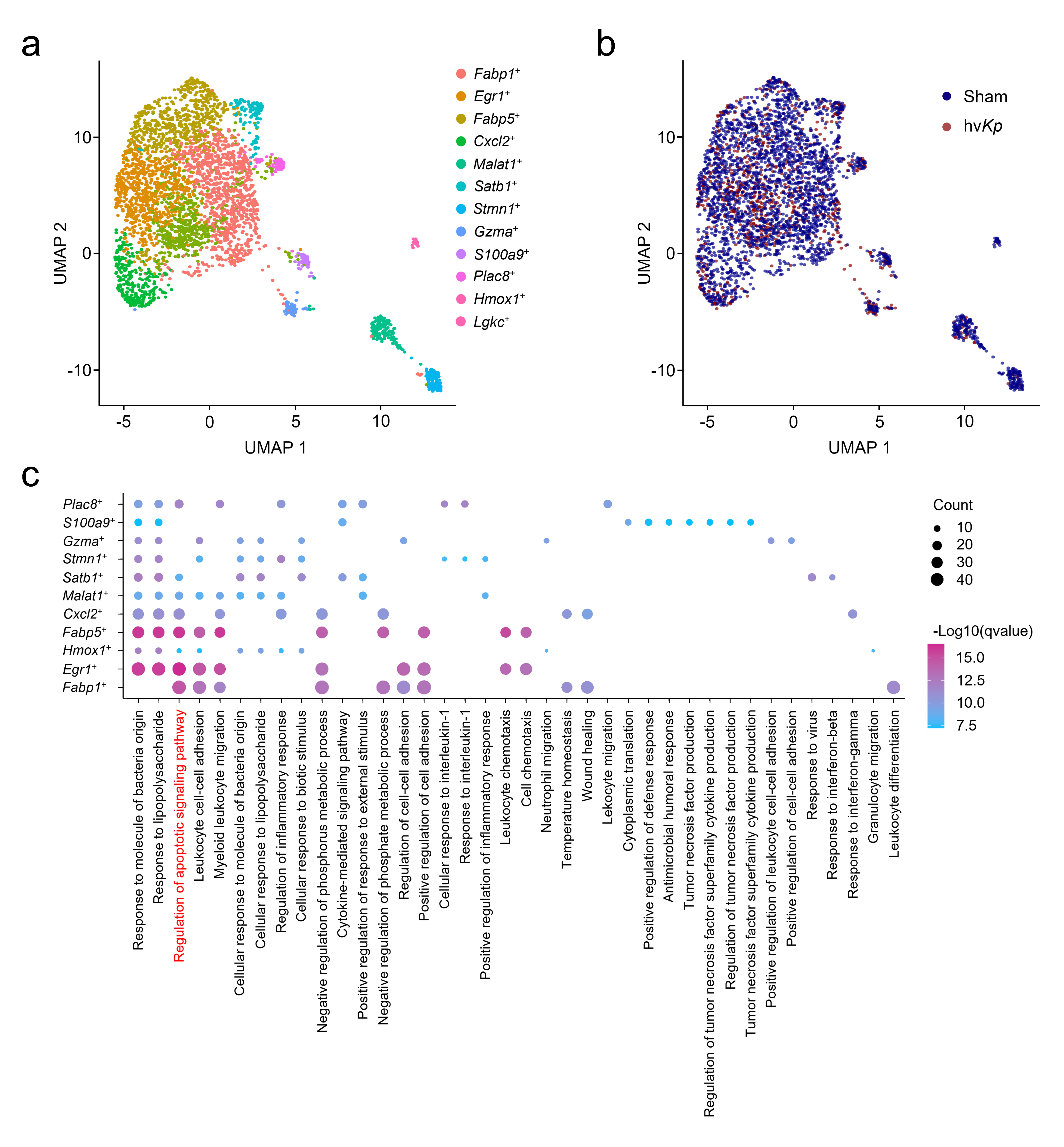
 Figure S1. Cellular landscape of AMs subsets in hv*Kp*-infected lungs.**

**(a)** Major clusters and respective cell-type assignments in UMAP. **(b)** Origins of cells with same embedding as in (a). **(c)** Go enrichment analysis of changing pathways in AMs.

**
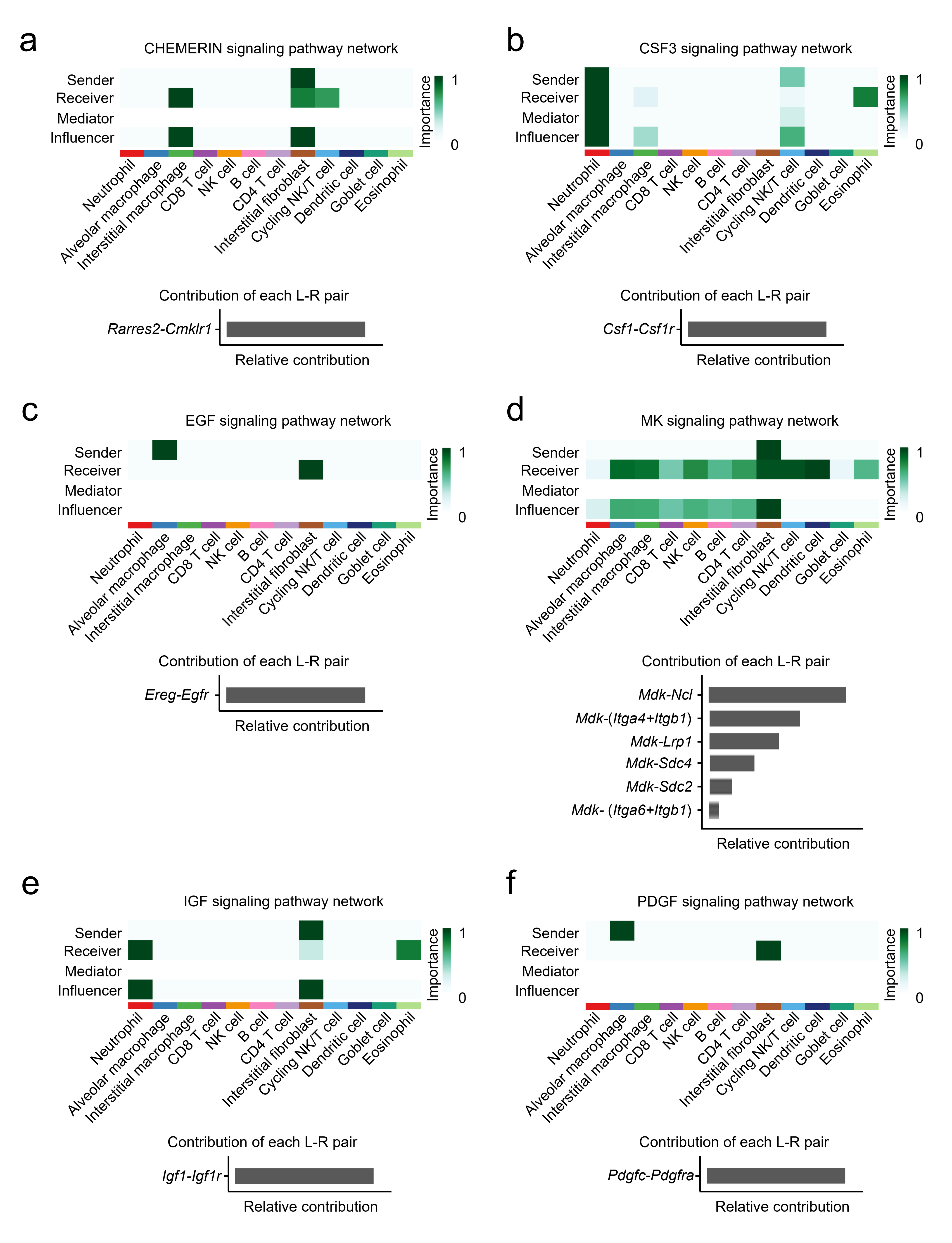
** **Figure S2. Cellular communication in *Kp* infected lungs.**

**(a)** The inferred CHEMERIN signaling network and relative contribution of each CHEMERIN ligand-receptor pair. **(b)** The inferred CSF3 signaling network and relative contribution of each CSF3 ligand-receptor pair. **(c)** The inferred EGF signaling network and relative contribution of each EGF ligand-receptor pair. **(d)** The inferred MK signaling network and relative contribution of each MK ligand-receptor pair. **(e)** The inferred IGF signaling network and relative contribution of each IGF ligand-receptor pair. **(f)** The inferred PDGF signaling network and relative contribution of each PDGF ligand-receptor pair.

**
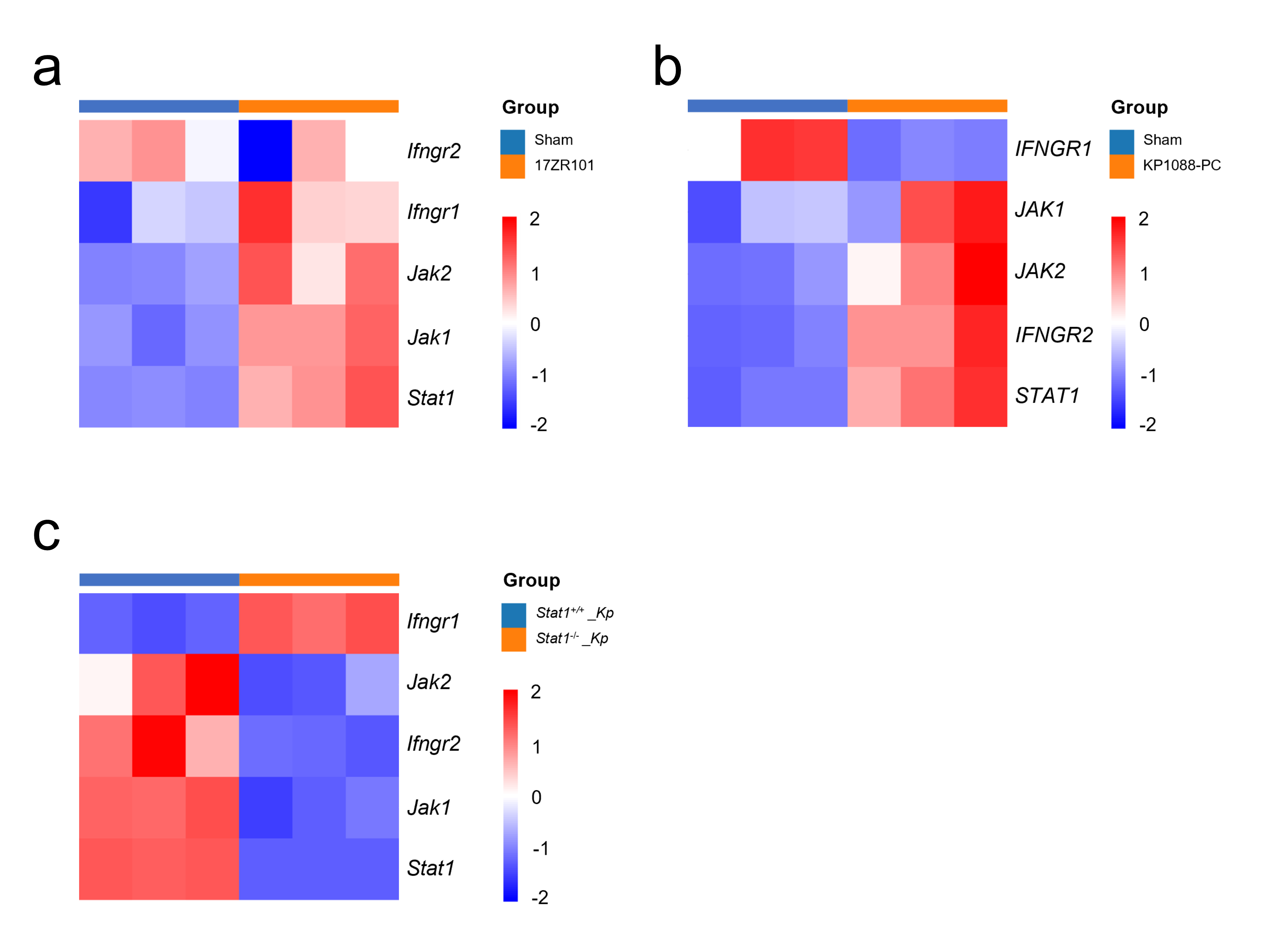
**

**Figure S3**. **mRNA expression of genes in JAK-STAT1 pathways.** Heatmap showed the differentially expressed genes in lung samples of mice **(a)**, THP-1 cells **(b)** and BMDMs **(c)** with *Kp* infection or sham control
