# Supplementary material for: Cytokine-driven PANoptosis of alveolar macrophages mediated by STAT1 underlies acute lung injury in hypervirulent Klebsiella pneumoniae infection
Source: mBio. 2026 Mar 24;17(5):e03958-25. doi: 10.1128/mbio.03958-25 (PMC13170300; doi:10.1128/mbio.03958-25)
Supplement: Table S2 — Primers for qPCR in this study. [file mbio.03958-25-s0003.docx]

| Gene | Strand | Sequence 5’-3’ |
| --- | --- | --- |
| M*-Nlrp3* | Forward | AGAAGAGACCACGGCAGAAG |
|  | Reverse | CCTTGGACCAGGTTCAGTGT |
| M*-Caspase-11* | Forward | ACAATGCTGAACGCAGTGAC |
|  | Reverse | CTGGTTCCTCCATTTCCAGA |
| M*-Il1b* | Forward | GGAGAGCCCTGGATACCAAC |
|  | Reverse | CAGGGTCCCAGACAGAAGTT |
| M*-Caspase-1* | Forward | AACAGAACAAAGAAGATGGCACA |
|  | Reverse | CCAACCCTCGGAGAAAGAT |
| M*-Gapdh* | Forward | CATCACTGCCACCCAGAAGACTG |
|  | Reverse | ATGCCAGTGAGCTTCCCGTTCAG |
| H*-GSDMD* | Forward | ATGAGGTGCCTCCACAACTTCC |
|  | Reverse | CCAGTTCCTTGGAGATGGTCTC |
| H*-CASP1* | Forward | GCTGAGGTTGACATCACAGGCA |
|  | Reverse | TGCTGTCAGAGGTCTTGTGCTC |
| H*-CASP7* | Forward | CGGAACAGACAAAGATGCCGAG |
|  | Reverse | AGGCGGCATTTGTATGGTCCTC |
| H*-RIPK1* | Forward | TATCCCAGTGCCTGAGACCAAC |
|  | Reverse | GTAGGCTCCAATCTGAATGCCAG |
| H*-GAPDH* | Forward | GAGTCAACGGATTTGGTCGT |
|  | Reverse | GACAAGCTTCCCGTTCTCAG |

**Table S2. Primers for qPCR in this study.**
